# Cellulose-Derived Gels for Topical Delivery: HPMC as a Functional Matrix for Porphyrinic Photosensitizers

**DOI:** 10.3390/gels11100824

**Published:** 2025-10-14

**Authors:** Emma Adriana Ozon, Andreea Mihaela Burloiu, Adina Magdalena Musuc, Gina Manda, Valentina Anuta, Cristina Elena Dinu-Pîrvu, Dumitru Lupuliasa, Ionela Victoria Neagoe, Mihai Anastasescu, Radu Petre Socoteanu, Irina Atkinson, Raul-Augustin Mitran, Daniela C. Culita, Rica Boscencu

**Affiliations:** 1 Faculty of Pharmacy, “Carol Davila” University of Medicine and Pharmacy, 6 Traian Vuia St., 020956 Bucharest, Romania; emma.budura@umfcd.ro (E.A.O.); andreea-mihaela.burloiu@drd.umfcd.ro (A.M.B.); valentina.anuta@umfcd.ro (V.A.); cristina.dinu@umfcd.ro (C.E.D.-P.); dumitru.lupuliasa@umfcd.ro (D.L.); rica.boscencu@umfcd.ro (R.B.); 2 Institute of Physical Chemistry—Ilie Murgulescu, Romanian Academy, 060021 Bucharest, Romania; manastasescu@icf.ro (M.A.); psradu@yahoo.com (R.P.S.); iatkinson@icf.ro (I.A.); rmitran@icf.ro (R.-A.M.); dculita@icf.ro (D.C.C.); 3 “Victor Babeş” National Institute of Pathology, 050096 Bucharest, Romania; ionela.neagoe@ivb.ro

**Keywords:** unsymmetrical porphyrin, hydroxypropyl methylcellulose, hydrogel formulations, HaCaT keratinocytes, A-431 human epidermoid cells

## Abstract

Hydroxypropyl methylcellulose (HPMC) is a biocompatible polymer widely used in topical formulations due to its suitable rheological behavior, film-forming capacity, and good compatibility with different active pharmaceutical ingredients. The present study demonstrates the potential of HPMC-based gels for dermal delivery of porphyrinic photosensitizers, aiming to enhance the efficiency of photodynamic therapy (PDT) in potential skin cancer applications. HPMC-based gel incorporating two previously synthesized porphyrinic photosensitizers, named 5,10,15,20-tetrakis-(4-acetoxy-3-methoxyphenyl) porphyrin (P2.1) and 5-(4-hydroxy-3-methoxyphenyl)-10,15,20-tris-(4-acetoxy-3-methoxyphenyl) porphyrin (P2.2), was developed and carefully characterized regarding its rheological behavior, texture, and in vitro activity. Fourier-transform infrared spectroscopy (FTIR), thermogravimetric analysis (TGA), X-ray diffraction (XRD), atomic force microscopy (AFM), fluorescence, and UV-Vis spectroscopy were carried out to evaluate the structural and morphological changes induced by the incorporation of the porphyrins in the HPMC gel matrix. The gels were subsequently evaluated by pharmacotechnical analysis, including pH (7.2 for both HPMC-P2.1 and HPMC-P2.2), viscosity, spreadability, texture profile analysis, and drug content uniformity. Rheological behavior confirmed the pseudoplastic behavior, suggesting a structured system with a gel-like consistency, while physical measurements demonstrated the stability and preserved functionality of the photosensitizers within the HPMC matrix. In vitro studies revealed an efficient cellular internalization of selected porphyrins into human epidermoid carcinoma cells, a critical requirement for topical PDT applications. The study highlights the capability of HPMC gels to serve as effective delivery platforms for porphyrin-based photosensitizers, supporting their application in localized skin cancer treatment through PDT.

## 1. Introduction

Each year, over one million individuals are diagnosed with skin cancer, making it one of the most widespread forms of cancer (more than 1.5 million new cases estimated in 2022 according to IARC/WHO), representing a significant problem for global health and the economy [1]. Topical photodynamic therapy (PDT) represents a modern non-invasive, selective, and promising therapeutic option for skin malignancies [2,3].

The therapeutic efficacy of photodynamic therapy (PDT) relies on the administration of a photosensitizer (PS), which, upon activation by specific wavelengths of light, generates reactive oxygen species such as singlet oxygen. These reactive species induce localized cytotoxicity, leading to tumor cell death while sparing surrounding healthy tissue [2,3,4]. The efficacy of PDT is strongly influenced by the delivery carrier used to transport and release the photosensitizer at the site of action. Topical application of photosensitizers offers distinct advantages for the treatment of cutaneous tumors by enabling localized drug delivery directly at the lesion site.

Porphyrins are frequently used in topical photodynamic therapy due to their strong selectivity for malignant cells, low cytotoxicity in the absence of light, and excellent photophysical properties, including a high absorption in the phototherapeutic range (600–800 nm). Additionally, they are effective in generating reactive oxygen species (ROS) [4,5]. However, the hydrophobic nature and poor aqueous solubility of many porphyrinic photosensitizers limit their bioavailability and therapeutic performance [4,5,6,7]. Structural modifications of the tetrapyrrole ring, such as introducing polarity-enhancing functional groups, have been shown to improve solubility and photodynamic therapy (PDT) performance, as demonstrated by the literature and supported by our previous studies [8,9,10,11,12,13,14]. As an alternative to structural modifications, pharmaceutical nanoformulation represents a promising strategy to optimize the targeted delivery and controlled release of the photosensitizer (PS) molecule within tumor tissues.

Carbohydrate-derived polymers, such as hydroxypropyl methylcellulose (HPMC), sodium alginate, and chitosan, have attracted significant interest as matrices for topical drug delivery. Their biocompatibility, biodegradability, and capacity to form hydrogels with adjustable mechanical and release properties make them particularly applicable [15,16,17,18,19].

Hydroxypropyl methylcellulose (HPMC), a semisynthetic cellulose derivative, has been widely used as a pharmaceutical excipient due to its excellent biocompatibility, film-forming ability, and capacity to create stable hydrogel matrices. Its non-ionic nature reduces the risk of irritation and facilitates compatibility with a broad range of active compounds. Furthermore, HPMC gels provide a moist environment that promotes drug permeation and sustained release, making them highly suitable for topical drug delivery applications [20].

The HPMC, as a hydrophilic polymer network, offers a favorable microenvironment that enhances porphyrin dispersibility by minimizing π–π stacking electrostatic interactions between porphyrin molecules and promoting physical separation of molecules within the gel matrix by inhibiting their aggregation [21]. Polymeric micelles have emerged as promising nanocarriers for hydrophobic porphyrin-based photosensitizers (PSs) in cancer theranostics, as they self-assemble from amphiphilic block copolymers into nanosized structures with hydrophobic cores that encapsulate PSs and hydrophilic shells that enhance stability and biocompatibility. The micellar environment helps minimize π–π stacking interactions between porphyrin molecules, preventing aggregation and improving dispersibility. Incorporating porphyrins into these micelles improves their solubility and stability, facilitating applications in photodynamic therapy (PDT) and imaging; porphyrins, when activated by light, generate reactive oxygen species (ROS) capable of selectively eliminating tumor cells, while their intrinsic fluorescence allows non-invasive imaging for diagnosis and treatment monitoring [22]. Furthermore, the polymer chains can form hydrogen bonds or hydrophobic interactions with the porphyrin derivatives, improving solubilization and stability [23]. This incorporation also allows for controlled, diffusion-based release of the active compounds, while the gel’s inherent mucoadhesive and rheological properties enhance skin retention and ensure close contact with the target tissue, allowing for optimal light exposure and reactive oxygen species (ROS) generation during PDT [24]. Combining these attributes contributes to a more uniform, sustained, and effective delivery of porphyrins for topical PDT applications, highlighting the potential of HPMC-based gels as advanced drug delivery platforms.

In this context, the present research aims to develop the incorporation of novel porphyrinic photosensitizers into HPMC-based gel formulations to enhance topical PDT for skin cancer. While our previous studies have reported the use of Carbopol or hydroxypropyl cellulose (HPC) gels for similar purposes [25,26], the unique physicochemical properties of HPMC may offer distinct advantages in terms of gel stability, rheological behavior, and drug release profiles.

The study presents the development and comprehensive physicochemical, rheological, and pharmacotechnical characterization of HPMC-based gels loaded with two porphyrin derivatives, 5,10,15,20-tetrakis-(4-acetoxy-3-methoxyphenyl) porphyrin (P2.1) and 5-(4-hydroxy-3-methoxyphenyl)-10,15,20-tris-(4-acetoxy-3-methoxyphenyl) porphyrin (P2.2), previously characterized in our works [10,27]. The novelty of the present study is related to the comparative approach regarding the influence of carbohydrate polymer matrices on physicochemical properties and cellular internalization into human epidermoid carcinoma cells, assessing their suitability for the design of efficient topical PDT formulations in skin carcinoma.

## 2. Results and Discussion

### 2.1. Physicochemical Characterization of the Gels

#### 2.1.1. FTIR Spectroscopy

**Figure 1:**
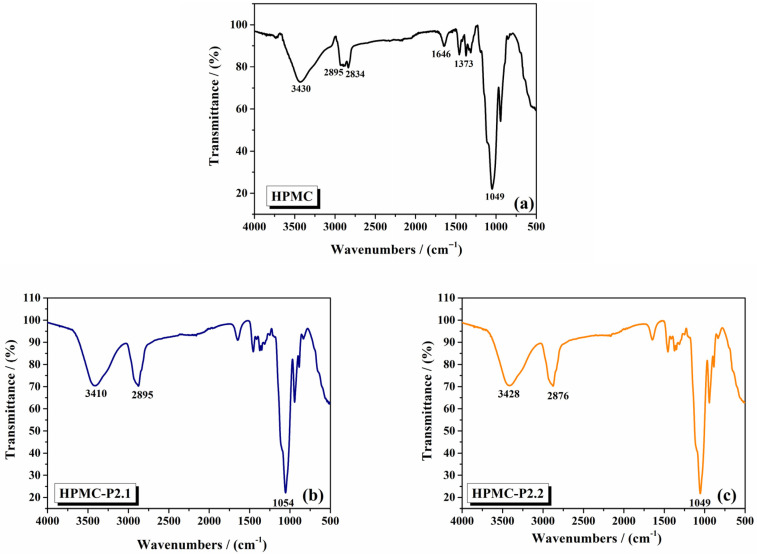
The FTIR spectra of (**a**) 10% HPMC gel, (**b**) HPMC-P2.1, and (**c**) HPMC-P2.2.

FTIR spectra of pure 10% HPMC, HPMC-P2.1, and HPMC-P2.2 gels were recorded in the 4000–400 cm^−1^ range. The spectra were analyzed to identify characteristic peaks and any shifts or new peaks indicative of interactions between HPMC and the porphyrins. The interactions between the polymer and the porphyrin derivatives were analyzed by comparing the spectrum of pure 10% HPMC gel with the spectra of HPMC-P2.1 and HPMC-P2.2 samples. The FTIR spectra of the analyzed hydrogels are displayed in Figure 1 (a—10% HPMC gel, b—HPMC-P2.1, c—HPMC-P2.2).

The FTIR spectrum of pure HPMC (Figure 1a) exhibited characteristic peaks at 3430 cm^−1^ (due to O-H stretching frequency), 2895 and 2834 cm^−1^ (due to C-H stretching), 1646 cm^−1^ (due to C=C stretching vibrations), 1373 cm^−1^ (assigned to C-H stretching and deformation vibrations), and 1049 cm^−1^ (due to C-O-C stretching) [28].

In the spectra of HPMC-P2.1 (Figure 1b) and HPMC-P2.2 (Figure 1c), notable changes were observed. A shift in the O-H stretching peak from 3430 cm^−1^ to 3410 cm^−1^ suggested hydrogen bonding between HPMC and P2.1. This downfield shift suggests a decrease in the bond energy due to interaction with the porphyrin derivative. The C-H stretching peak at 2895 cm^−1^ broadens and shifts slightly, which is a confirmation of the interactions between the HPMC matrix and P2.1. The peak at 1049 cm^−1^ becomes less intense and slightly shifted to 1054 cm^−1^, indicating possible interactions involving the ether linkages of HPMC and the porphyrin derivative. These spectral changes are indicative of possible hydrogen bonding between HPMC and P2.1, leading to structural modifications within the gel matrix. Similar shifts in the O-H stretching region were observed, along with characteristic peaks for the methoxy and acetoxy groups of P2.2 (Figure 1c). The O-H stretching peak shifts from 3430 cm^−1^ to 3428 cm^−1^, similar to the shift observed with P2.1, indicating interactions between HPMC and P2.2. These shifts confirmed the successful incorporation of P2.2 into the HPMC gel. The C-H stretching peak at 2895 cm^−1^ shows broadening and slight shifting (2876 cm^−1^), indicating interactions between HPMC and P2.2. The intensity of the peak at 1049 cm^−1^ increases, suggesting interactions involving the ether linkages of HPMC and P2.2. These observations confirm that P2.2 is successfully incorporated into the HPMC gel, interacting with the polymer matrix and causing structural changes. The literature indicates that HPMC displays a broad O-H stretching band around 3400 cm^−1^, typical for hydroxyl groups in cellulose derivatives [29]. Shifts in this region upon incorporating drugs or other compounds often indicate hydrogen bonding. The broadening and shifting of the C-H stretching peak and changes in the C-O-C stretching peak intensity suggest interactions between HPMC and the porphyrins. In the literature, there are reports of similar modifications in these regions for polymer–drug systems, attributed to interactions affecting the polymer’s structural features [30]. These results corroborate previous observations, confirming the successful incorporation of porphyrins into the HPMC matrix and their interactions.

The FTIR analysis of 10% HPMC gels with porphyrin derivatives P2.1 and P2.2 reveals distinct structural changes, indicating possible interactions between the polymer matrix and the porphyrin compounds. Shifts in the O-H stretching region and modifications in the C-H and C-O-C stretching regions suggest successful incorporation and molecular interaction between the HPMC polymer and the porphyrins.

#### 2.1.2. X-Ray Diffraction Analysis

The X-ray diffractograms were analyzed to determine the crystalline or amorphous nature of the samples and to identify any structural changes due to the incorporation of porphyrins.

**Figure 2:**
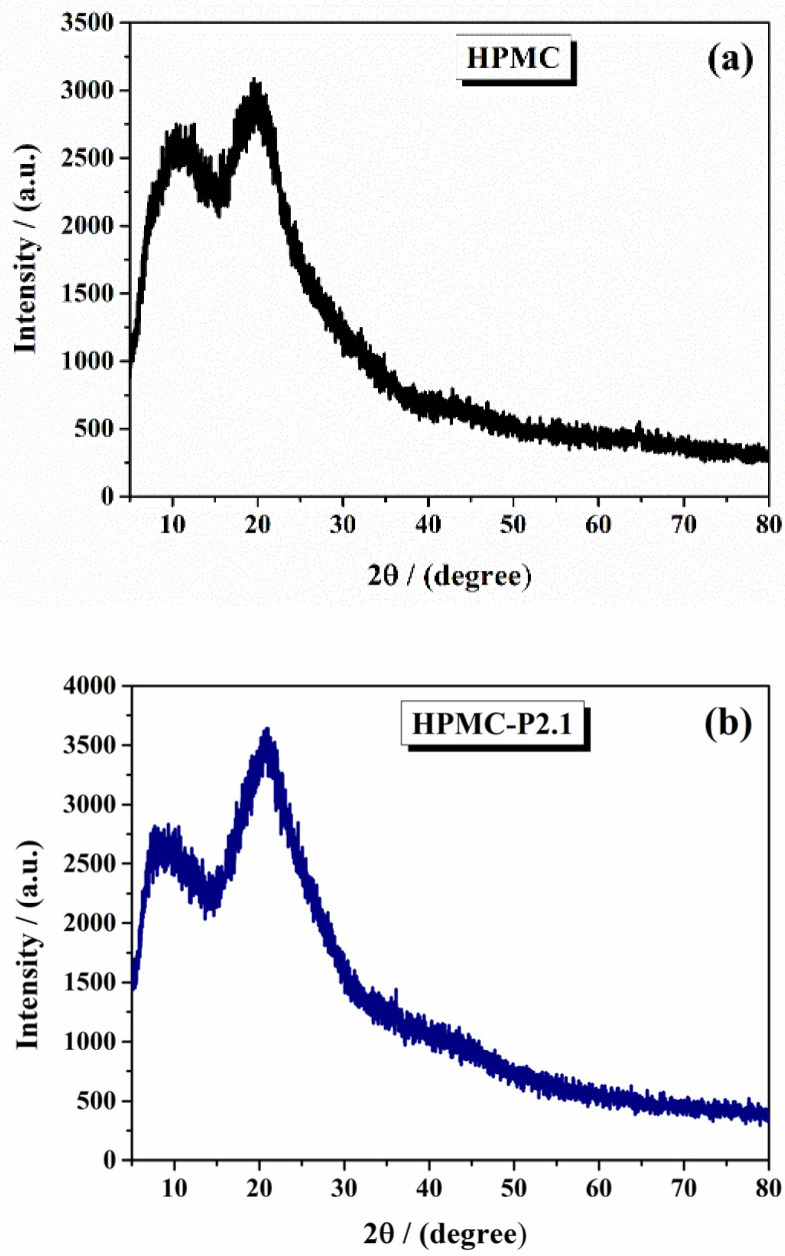

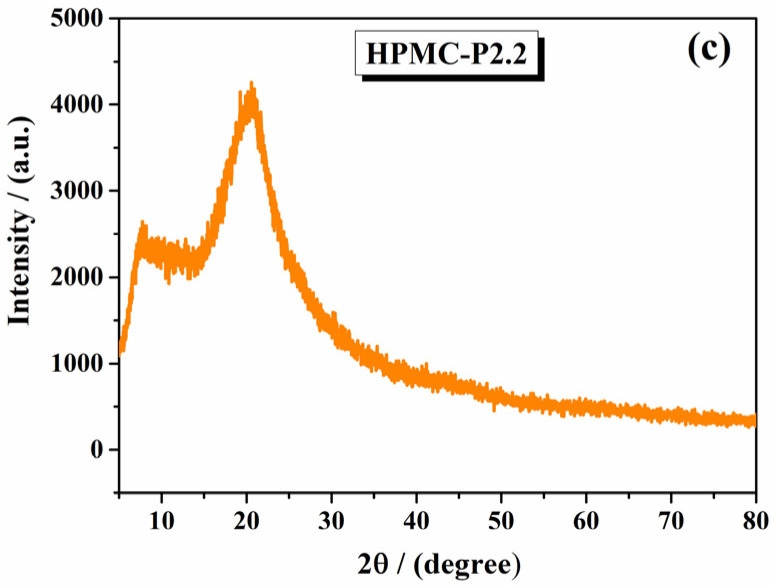
The XRD diffractograms of (**a**) 10% HPMC gel, (**b**) HPMC-P2.1, and (**c**) HPMC-P2.2.

The XRD pattern of pure HPMC (Figure 2a) showed a broad, diffuse peak centered around 20°, characteristic of an amorphous polymer. In contrast, the XRD patterns of HPMC-P2.1 and HPMC-P2.2 revealed significant changes. The diffractogram of HPMC-P2.1 (Figure 2b) displayed an increase in the intensity of the peak at 20°, indicating a slight modification in the amorphous structure of HPMC due to the incorporation of P2.1. These peaks corresponded to the ordered arrangement of the porphyrin molecules within the HPMC matrix. Similar to HPMC-P2.1, HPMC-P2.2 (Figure 2c) showed a higher increase in the peak intensity, suggesting that P2.2 also induced crystallinity in the otherwise amorphous HPMC. The differences in peak positions and intensities between P2.1 and P2.2 highlighted the distinct structural impact of each porphyrin derivative.

XRD analysis demonstrated that the incorporation of porphyrin derivatives P2.1 and P2.2 into HPMC gels resulted in the formation of some crystalline regions within the amorphous polymer matrix. The literature frequently reports that the incorporation of drug molecules into polymer matrices can induce crystallinity, particularly when the drug has a propensity to form ordered structures, and some of them cause less crystallinity [31,32]. The sharp peaks observed in the XRD patterns of HPMC-P2.1 and HPMC-P2.2 are consistent with this phenomenon, confirming that both porphyrin derivatives induce partial ordering within the HPMC gel.

#### 2.1.3. Thermal Analysis

**Figure 3:**
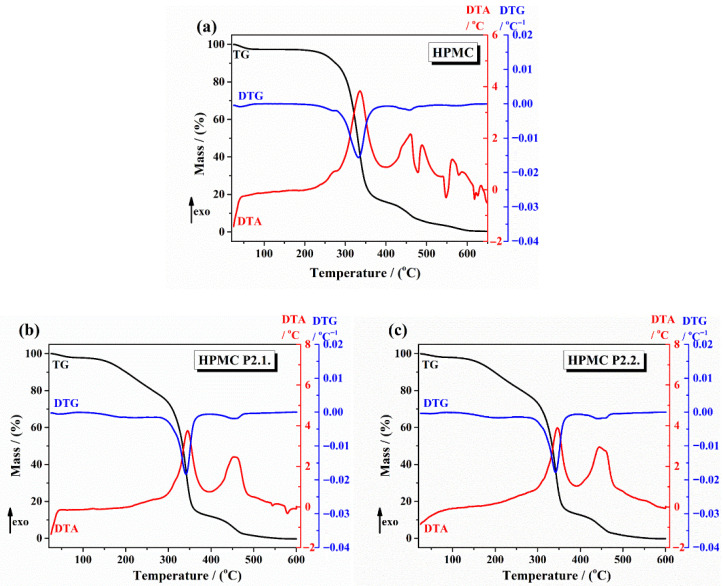
The TG/DTG and DTA curves of (**a**) 10% HPMC gel, (**b**) HPMC-P2.1, and (**c**) HPMC-P2.2.

The thermal TGA curves are displayed in Figure 3 ((a) 10% HPMC gel, (b) HPMC-P2.1, (c) HPMC-P2.2). The thermal data are shown in Table 1.

For 10% HPMC gel (Figure 3a), the first mass loss below 100 °C is attributed to the loss of absorbed water. HPMC exhibited a major weight loss starting around 250 °C, indicating the decomposition of the polymer. HPMC-P2.1 showed a two-step weight loss, with the first step similar to HPMC, attributed to the loss of water. The first loss started around 200 °C (decomposition of P2.1) and the second step around 260 °C (decomposition of HPMC), suggesting an enhanced thermal stability due to the porphyrin. HPMC-P2.2 displayed a similar two-step weight loss pattern, with initial decomposition starting at 210 °C, indicating that the incorporation of P2.2 also improves the thermal stability of the HPMC gel.

Thermal analysis via TGA revealed that the incorporation of porphyrin derivatives P2.1 and P2.2 significantly affects the thermal properties of HPMC gels. The altered decomposition patterns indicate improved thermal stability and potential interactions between HPMC and the porphyrins, which are essential for the development of stable and effective PDT formulations.

The two-step degradation patterns observed in TGA for HPMC–drug systems have also been reported in the literature, where the first step corresponds to the degradation of the drug and the second to the degradation of polymer matrix [33,34].

Our thermal analysis results align well with these studies, suggesting that the porphyrins not only interact with HPMC but also enhance its thermal stability.

FTIR, XRD, and thermal analyses of both 10% HPMC gels containing porphyrin derivatives P2.1 and P2.2 offer comprehensive insights into their physicochemical characteristics. These studies elucidate the structural changes, molecular interactions, and thermal behaviors within the gel matrices, which are essential for designing effective photodynamic therapy (PDT) formulations targeting malignant and non-malignant skin conditions. The physicochemical profiles of these gels demonstrate their promising potential for topical PDT applications.

The observed crystallinity and modified thermal stability suggest that the HPMC gels are more stable and could provide a controlled porphyrin release. These observed structural modifications and interactions ensure that the porphyrin derivatives are well-incorporated in the HPMC gel matrix, facilitating targeted, effective delivery. The strong interactions between the HPMC and the porphyrins support their potential efficacy in treating various diseases through photodynamic treatment.

#### 2.1.4. AFM Results

**Figure 4:**
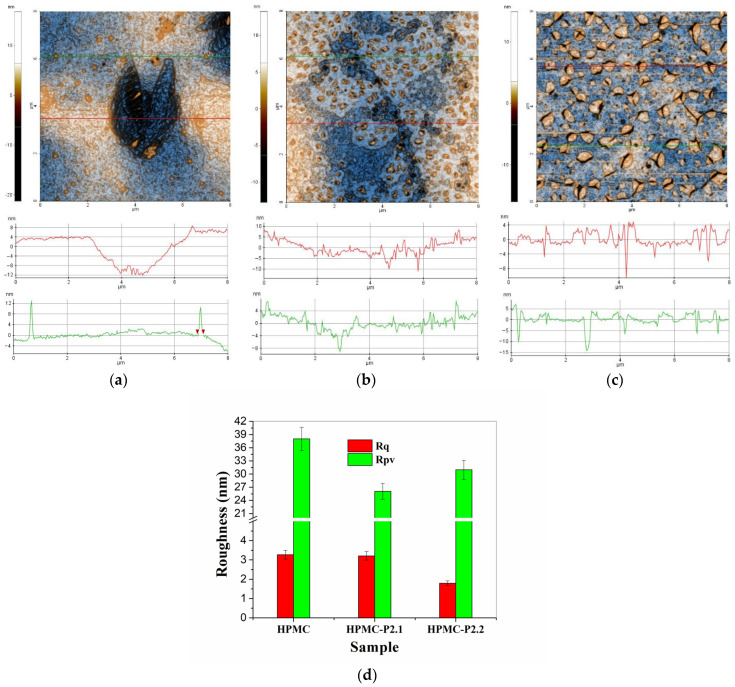
Two-dimensional topographic AFM images, enhanced contrast, of the 10% HPMC (**a**), HPMC-P2.1 (**b**), and HPMC-P2.2 (**c**) samples at a scale of 8 µm × 8 µm. Two characteristic surface profiles (green and red scan lines) are shown below each AFM image. Histogram of roughness parameters (RMS roughness—Rq, peak-to-valley parameter—Rpv) for 10% HPMC, HPMC-P2.1, and HPMC-P2.2 samples at 8 µm × 8 µm scale (**d**).

In Figure 4 are presented the two-dimensional (2D) AFM images, topographic mode with enhanced contrast, of hydrogels based on hydroxypropyl methylcellulose (10% HPMC, HPMC-P2.1, and HPMC-P2.2), recorded at a scale of 8 µm × 8 µm. Below each AFM image are exemplified characteristic profile lines (constituent scan lines of the presented images, indicated by green and red lines in AFM images).

In Figure 4a is presented the surface topography of the hydrogel of hydroxypropyl methylcellulose (10% HPMC) without the addition of porphyrins. It can be noted that the surface of the HPMC gel tends to form superficial cavities with a depth of 15–16 nm (see the dark area in the center of Figure 4a and the upper right corner), the diameter being about 3 microns. Outside these cavities, the gel surface is continuous, relatively compact, and presents particles of about 2–300 nm, randomly arranged (see the two profile lines below the AFM image—Figure 4a).

The HPMC gel is characterized by an RMS roughness (Rq) of 3.3 nm and a peak-to-valley parameter (Rpv) of about 38.0 nm. The incorporation of porphyrin P2.1 into the polymer gel with 10% HPMC modifies the appearance of the sample. Its main characteristic belong to a population of particles (yellow clusters) with a diameter of 1–300 nm (the presence of these particles makes the profile lines in Figure 4b have a “jagged” appearance). In the vicinity of each particle, there are small depressions (“dark” shadows), suggesting on the one hand that these particles (protuberances) are formed by drying the gel at ambient temperature and on the other hand that the incorporation of porphyrin P2.1 into HPMC modifies its viscosity. The HPMC-P2.1 sample is characterized by an RMS roughness (Rq) of 3.2 nm and a peak-to-valley parameter (Rpv) of about 26.0 nm. The incorporation of porphyrin P2.2 into the HPMC polymer gel (HPMC-P2.2 sample) has the effect of increasing the dimensions of surface “particles”. These particles can be seen as small agglomerations of material, small “clusters”, formed either from the parent material (gel + porphyrin) or by the segregation of a constituent as a result of the modification of the gel viscosity following the incorporation of the porphyrin. The depressions (“nano-pits”) observed in the case of the HPMC-P2.1 sample, in this case, take the form of micro-cracks that furrow these clusters (particles) or are located at their edge (thus suggesting differences in the surface tension of the two materials: HPMC-P2.1 and HPMC-P2.2). For this reason, the differences on the vertical axis of the profile lines in Figure 4c are more pronounced compared to those observed in Figure 4b. The HPMC-P2.2 sample is characterized by an RMS roughness (Rq) of 1.8 nm and a peak-to-valley parameter (Rpv) of about 30.1 nm. The corrugation parameters of the series of hydrogels on the hydroxypropyl methylcellulose base are presented in the form of histograms in Figure 4d.

**Figure 5:**
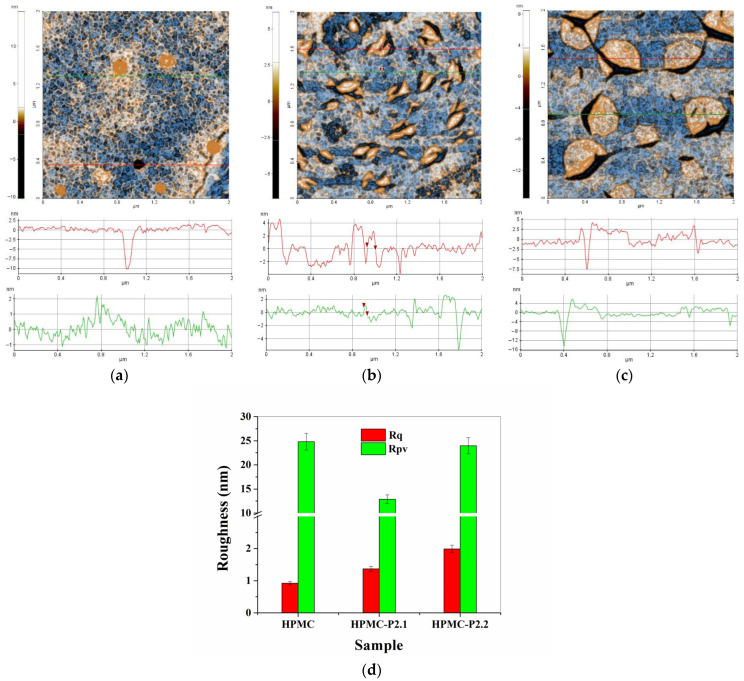
Contrast-enhanced two-dimensional topographic AFM images of the 10% HPMC (**a**), HPMC-P2.1 (**b**), and HPMC-P2.2 (**c**) samples at a scale of 2 µm × 2 µm. Two characteristic profiles (scan lines) are shown below each AFM image. Histogram of roughness parameters (RMS roughness—Rq, peak-to-valley parameter—Rpv) for 10% HPMC, HPMC-P2.1, and HPMC-P2.2 samples at 2 µm × 2 µm scale (**d**).

By scanning at a smaller scale, the morphological differences between the hydroxypropyl methylcellulose-based hydrogels are more clearly highlighted (Figure 5). For example, Figure 5a highlights the fact that the HPMC sample has a nano-porosity given by the presence of small depressions (~1–2 nm), well highlighted by the green profile line in Figure 5a. The red profile line highlights the presence of a pit of about 10 nm in depth. The presence of material protuberances (particle appearance) with diameters of about 100–150 nm can also be observed. At this scale (2 µm × 2 µm), the HPMC sample is characterized by an RMS roughness (Rq) of 0.9 nm and a peak-to-valley parameter (Rpv) of about 24.8 nm. Looking at the HPMC-P2.1 sample (Figure 5b), it can be seen that the protuberances observed at a large scale in Figure 4b are elongated, having a vermicular appearance. The small “pits” (2–4 nm deep) in the vicinity of these vermicular formations (with diameters of up to 100 nm) appear due to the tension of the material that gathers in the protuberances, leaving a material deficit in the immediate vicinity. The HPMC-P2.1 sample is characterized by an RMS roughness (Rq) of 1.4 nm and a hill–valley parameter (Rpv) of about 12.9 nm. Between these formations, there are areas morphologically similar to the parent HPMC gel (nanoporous).

Finally, for the HPMC-P2.2 sample (Figure 5c), it can be observed that the areas with segregated material, with a semi-spherical appearance, have a well-relieved edge but with a height of a few nm (having the appearance of flattened shells). In the immediate vicinity or furrowing these formations, some grooves give the appearance of micro-cracks, with depths of 10–20 nm (thus contributing more to the corrugation parameters). Between these formations (the “flattened shell” type), the imprint of the morphological nanoporosity of the HPMC parent gel can be recognized. The HPMC-P2.2 sample is characterized by an RMS roughness (Rq) of 1.9 nm and a hill–valley parameter (Rpv) of about 23.9 nm. The corrugation parameters of the series of hydrogels based on hydroxypropyl methylcellulose, estimated at a scale of 2 µm × 2 µm, are grouped in the form of histograms in Figure 5d.

#### 2.1.5. UV-Vis and Fluorescence Spectroscopy

**Figure 6:**
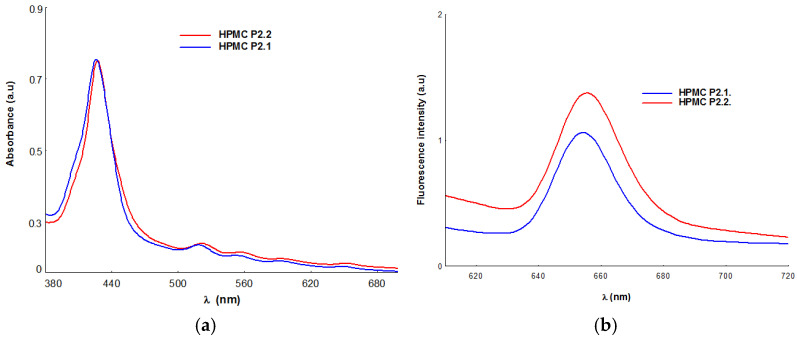
Absorption (**a**) and fluorescence (**b**) spectra of HPMC gels with porphyrins (P2.1 and P2.2).

The spectral analysis applied to the gel formulations obtained by integrating porphyrins P2.1 and P2.2 into the HPMC polymer matrix revealed the maintenance of the spectral profile corresponding to the porphyrin structures, with the maintenance of the absorption and emission maxima in the spectral range relevant for the photodynamic treatment applicability of the obtained systems [2,35,36]. The values associated with the absorption and fluorescence maxima of the HPMC gels that include the P2.1 and P2.2 porphyrins are presented in Table 2. In Figure 6, the absorption and emission spectra of P2.1 and P2.2 in the HPMC polymer matrix are presented for example.

From a spectral point of view, the samples retain the specific profile of tetrapyrrolic porphyrin-type structures, described by an intense signal positioned at approximately 426 nm (Soret band) and four other spectral signals assigned to Q bands, with weaker intensity, which are identified in the spectral range of 518–656 nm [37,38].

UV-Vis spectral analysis confirmed the presence of porphyrin structures P2.1 and P2.2 in the HPMC polymeric matrix and the stability of porphyrins in this formulation variant, all spectral characteristics remaining unaltered (Figure 6). The two porphyrin structures were evaluated spectrally and biologically in a previous study [27], and by comparison with spectral parameters associated with P2.1 and P2.2 in solutions with PEG 200 solvent or their dilutions with phosphate-buffered solution (PEG 200/PBS = 1/1000), the present study did not reveal significant changes in the spectral profile responsible for the photosensitizing activity of the porphyrin. Related to the emission properties of P2.1 and P2.2, the analysis of the investigated samples highlights the presence of a strong fluorescent signal in the spectral range of 654–656 nm, a signal that confirms the potential applicability in PDT of gel formulations containing P2.1 and P2.2 included in the HPMC-type polymeric matrix. The study highlights small differences between the emission maxima of solutions with P2.1 and P2.2 dissolved in PEG or PEG/PBS and of the P2.1–HPMC and P2.2-HPMC systems, and it demonstrates that HPMC does not influence the fluorescent properties of the two photosensitizers. The spectral evaluation of the two formulas obtained by integrating P2.1 and P2.2 in HPMC-type matrices confirms the presence of the photosensitizer in the polymer matrix with the maintenance of the spectral profile and potential applicability in PDT at the cutaneous level.

### 2.2. Pharmacotechnical Evaluation of the Porphyrin Gels

The resulting hydrogels had no visible air bubbles and were homogeneous, sticky, translucent, and slightly pink. The 10% HPMC gel had a density of 0.976 g/mL, while the porphyrin gels had increased densities of 1.26 g/mL for HPMC-P2.1 and 1.15 g/mL for HPMC-P2.2.

#### 2.2.1. Wet Gel Evaluation

##### pH Determination

The base gel’s pH was 6.5 but was raised to 7.2 for both HPMC-P2.1 and HPMC-P2.2 by the addition of the porphyrins. The two porphyrins increased the pH significantly, although the values were the same in both formulations. When applied to the skin, the pH values of the two HPMC-gel incorporated porphyrin formulations obtained ensure good tolerance and no irritability [39]. Punitha et al. [40] demonstrated the influence of pH on the structural and physicochemical properties of HPMC hydrogels. It was found that the most suitable mechanical characteristics were achieved at a neutral pH. The acidic pH significantly reduced the viscosity due to the increased ionic strength, the higher concentration of HPMC macromolecules, and the likelihood of reduced polymer–polymer interaction. On the contrary, as the HPMC molecules are considerably stretched in the basic pH environment, they can interact with each other, leading to polymer–polymer interactions.

Geetha and Rakkappan [41] also found that the orientation and structural conformation of HPMC molecules are altered by pH changes, resulting in weak and strong interactions between water–polymer and polymer–polymer molecules, respectively. Based on the results obtained, it can be assumed that the two porphyrins have a good influence on the hydrogel properties and ensure adequate stability and tolerability in the final hydrogels.

##### Spreadability

**Figure 7:**
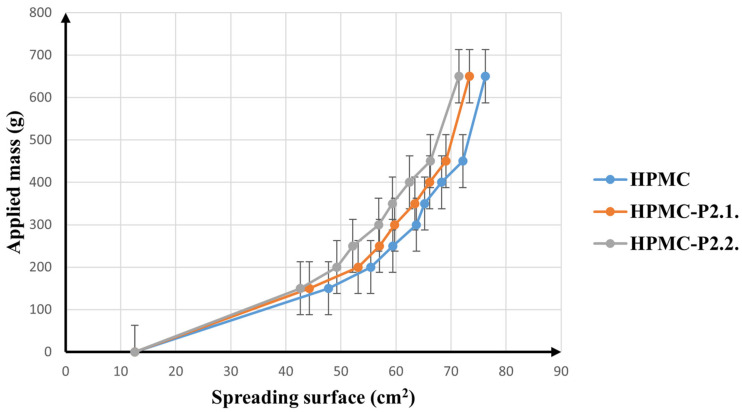
The spreading behavior of the hydrogels (10% HPMC, HPMC-P2.1, and HPMC-P2.2).

Figure 7 illustrates how the spreading surface varies with the applied mass.

All three samples show similar behavior, with a clear increase in extensibility after the initial load of 300 g; then, the spreading surface expands less noticeably up to 450 g and remains constant at weights above 450 g. After incorporation of the two porphyrin solutions into the hydrogel matrix, only a slight decrease in the extension areas was observed, proving that none of the porphyrins significantly altered the spreading ability of the HPMC gel. This can easily be explained by the fact that only a small amount of solution was added to the HPMC gel network. Nevertheless, the presence of a more liquid phase in the system should lead to an increase in spreadability, while a minor decrease was registered in this case. The increased cohesiveness in the porphyrin-loaded gels can be explained by the influence of PEG 200, which was used to dissolve them. PEG 200 acts as a plasticizant in the formulation and has been shown to form physical cross-links with HPMC gels [42,43]. Also, Giang Ha et al. [44] proved that the addition of PEG to an HPMC-based hydrogel significantly improves the cohesive strength of the gel matrix. Similarly, Khanum et al. [45] have shown how the cross-linker greatly increases the viscosity of HPMC gels, which ultimately leads to reduced spreadability.

Ferrari et al. [46] established a long time ago that the gelling power of HPMC hydrogels is associated with the cross-linking degree in the polymer matrix.

The findings demonstrate that the hydrogels will most likely spread quickly and uniformly throughout the applied skin region.

##### In Vitro Adhesion Ability

A slight difference was found between the adhesive properties of the hydrogels tested. The registered tensile strength values were 43 g/cm^2^ for the base gel, 41 g/cm^2^ for HPMC-P2.1, and 47 g/cm^2^ for HPMC-P2.2. Certainly, PEG 200 influences the cohesiveness of the systems, but it is clear that the porphyrins also influence the adhesive strength of the gel matrix. P2.1 led to an insignificant decrease in adhesion ability, while P2.2 seemed to form new bonds in the system, leading to an enhancement in adhesion performance, which may explain the decrease in the spreading properties of the HPMC-P2.2 hydrogel. Pan et al. [47] demonstrated that adhesiveness is enhanced by the intermolecular interactions between the gel-forming polymer and the incorporated active ingredient. The length of time for which the hydrogel and the skin remain in contact increases with the adhesive strength, so that it can be assumed that HPMC-P2.2 will have a higher residence time on the skin surface than HPMC-P2.1. Also, Joshi [48] found that the hydrophobic associations essentially control the strength of the gel, supporting the idea that the adhesiveness of hydrogels is shaped by the intermolecular links and the structural features of the matrix. Meanwhile, Chanoong et al. [49] stated that the amphiphilic properties of HPMC induce an adaptive interaction mechanism in water and that the ratio between hydrophilic and hydrophobic fractions is responsible for its effective binder character in the presence of water. 

##### Rheology Measurements

**Figure 8:**
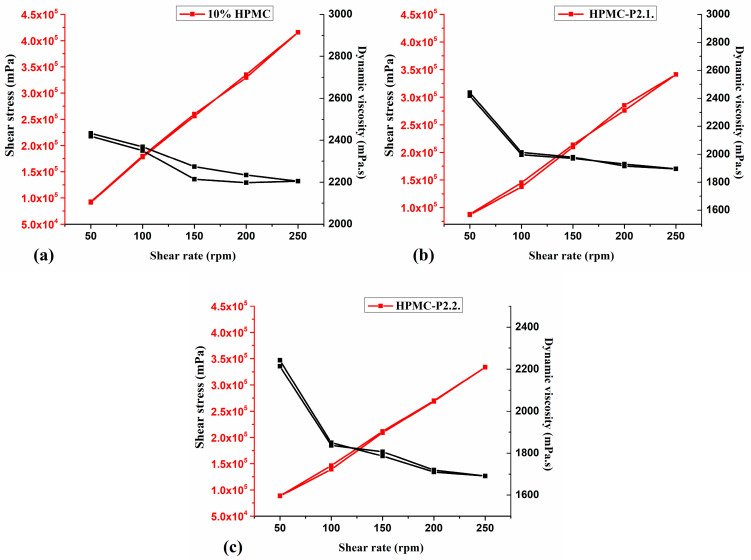
Rheological analysis (**a**) 10% HPMC, (**b**) HPMC-P2.1, and (**c**) HPMC-P2.2 samples.

The rheological data are represented in Figure 8.

The tested hydrogels exhibited the pseudoplastic behavior typical of gelled systems, where an increase in the shear rate leads to a decrease in dynamic viscosity and an increase in shear stress. All samples showed thixotropic characteristics, with similar shear stress and dynamic viscosity in both the upwards and downwards directions of the shear rate (Figure 8). The viscosity of the HPMC gel (Figure 8a) was slightly reduced by the addition of porphyrins to the gel matrix, which shows that the structure of the system and, consequently, the viscoelastic behavior and flowability were not changed significantly.

The results are consistent with the findings of Vanti et al. [50] on the shear-thinning properties of HPMC hydrogels, with viscosity decreasing with increasing speed, indicating a non-Newtonian flow rate. Szulc-Musioł et al. [51] explained the pseudoplastic flow behavior based on the polymer particles’ movement in the flow direction, reducing the viscosity of the system due to the increased shear stress.

Several studies [52,53,54] have revealed that PEG with different molecular weights exhibits a plasticizer effect on hydrogel networks, resulting in a reduction in the viscosity of the systems. Based on these observations, the slight decrease may diminish the dynamic viscosity of HPMC-P2.1 (Figure 8b) and HPMC-P2.2 (Figure 8c). This is due to the influence of PEG 200 and not necessarily to the porphyrins’ binding.

The spreading, adhesion, and viscoelastic properties of the HPMC base gel and the HPMC-P2.1 and HPMC-P2.2 hydrogels are similar and correlate strongly with each other. This shows that the addition of the PEG 200 solutions of the porphyrins to the HPMC matrix has only a minor influence, which proves that no new strong bonds are formed, but only physical interactions, probably through H-bonds.

#### 2.2.2. Dry Gel Evaluation

The films have a thickness of approximately 0.06 ± 0.003 mm, and there are no discernible differences between the loaded and base gels. Since the thickness of the film is related to the active ingredient’s concentration and the ability of the polymer to bioadhere, consistency is essential [55,56]. According to Yu et al. [57], the thickness of the films has a major influence on their release rate, adhesion, and mechanical properties. Dong et al. [58] established that in order to guarantee a controllable film thickness, adequate mechanical properties are essential, all of which depend on the changes in the water environment.

The values of the mechanical strength were 0.85 kg/mm^2^ for HPMC-P2.1, 0.79 kg/mm^2^ for HPMC-P2.2, and 0.71 kg/mm^2^ for the HPMC base gel. It can be noted that the incorporation of porphyrins into the HPMC matrix greatly influences the resistance of the film by enhancing it. This effect may also be due to the presence of the plasticizer, PEG 200, in the formulation.

According to various reports [59,60], the addition of plasticizers to a hydrogel is a useful way to reduce brittleness, as they reduce the bonding between the molecules of the polymers and create a new hydrogen bond with the polymers.

On the other hand, when porphyrins are added to the matrix, the elongation of the HPMC gel increases, from 12% for the HPMC gel to 15% for HPMC-P2.1 and 14% for HPMC-P2.2. Imran et al. [61] stated that to improve the molecular mobility and intermolecular spacing of the polymer chains in films, a plasticizer is required. This makes the films more flexible and stretchable. Compatibility with the polymers, the right amount of plasticizer, and the amount of free hydroxyl groups in a plasticizer are important factors regarding the ability to generate a high-quality film. Therefore, the type of plasticizer and the right concentration are essential for its effective application under different conditions.

Müller et al. [62] also showed that plasticizers often increase water permeability, so they must be used in moderation to produce films with better flexibility, thickness, and transparency without significantly reducing their mechanical strength or mass transfer barrier properties.

The moisture content was found to be similar for the three gels: 7.98% for the HPMC base, 8.11% for HPMC-P2.1, and 9.08% for HPMC-P2.2.

According to Ghadermazi et al. [63], HPMC films tend to form cross-links with water, and the addition of plasticizers in the structure can also increase the moisture content, as new hydrogen bonds are formed with the polymer chains, creating more space between the chains for water absorption.

The results are consistent with those of Zhang et al. [64], who demonstrated that the properties of HPMC films were significantly affected by the addition of various plasticizers, including glycerol, polyethylene glycol, and 1,2-propylene glycol. These plasticizers contributed to the lamellar structure, reduced tensile strength, increased the degree of crystallization, and increased the elongation at break of pure HPMC.

**Figure 9:**
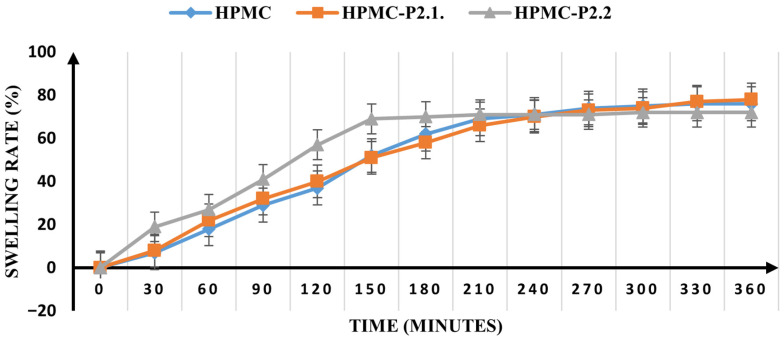
The swelling rate of the studied samples.

The swelling rate of the samples was tested over 6 h, and the results are shown in Figure 9.

HPMC-P2.1 retains the swelling properties of the HPMC base gel, reaching maximum absorption after 210 min (66% and 69%, respectively), while HPMC-P2.2 reaches maximum absorption after 150 min (69%), indicating a much faster moisture absorption. However, HPMC-P2.1 still seems to be able to absorb moisture after 360 min, but to a much lesser extent. In the meantime, HPMC-P2.2 attains a plateau at the higher absorption point, after which no more swelling is observed. The results show that, in addition to the influence of PEG 200 on the hydrogel structure, porphyrins also cause changes that are responsible for the entanglement of the polymer chains. According to Garg and Kumar [65], the diffusivity of water into the polymer is an important factor that has a significant effect on the initial swelling of films.

The polymer must be hydrated to expand and form a sufficiently large macromolecular network and to create the chain mobility that improves the polymer’s ability to deliver the active ingredients it contains to the skin surface. The swelling capacity, which exposes the bioadhesive sites for hydrogen bonding, enables mechanical entanglement [66]. The effect of the porphyrins on the swelling properties of the HPMC matrix is mainly determined by the substituted groups of the polymer, since the integrity of the swollen film is significantly influenced by the hydroxyl group present in the molecules [67]. The porphyrins are dissolved in PEG 200, and the solution creates a swelling force within the matrix of the hydrogel, resulting in a highly porous matrix. The penetration of water leads to deterioration in the integrity of the polymer network, which impairs the structural resilience of the swelling matrix and leads to erosion of the hydrogel structure [68].

### 2.3. In Vitro Studies

Considering that PS-HPMC gels are intended to be used for local PDT in skin carcinoma, a preliminary in vitro study was performed on A-431 human epidermoid cells, addressing the biocompatibility of 10% HPMC gels and the uptake by these cells of the P2.1 and P2.2 porphyrins formulated in HPMC gel.

#### 2.3.1. Biocompatibility of the HPMC Gel

**Figure 10:**
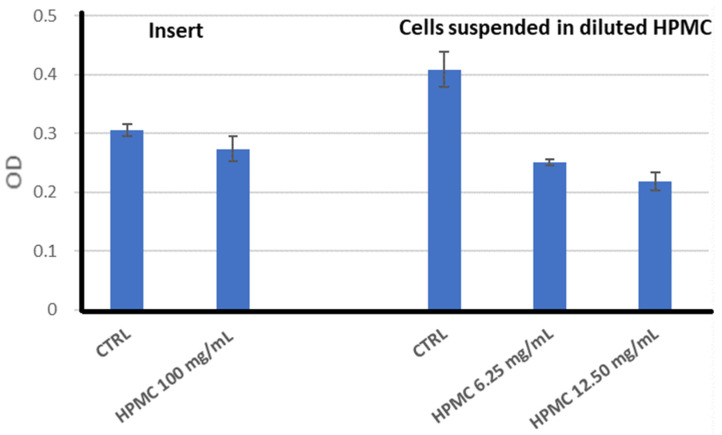
MTS reduction by A-431 human epidermoid carcinoma cells exposed to HPMC in the following settings: (i) gel added in inserts; (ii) cells suspended in HPMC diluted with culture medium by mechanical homogenization. Results are presented as mean OD (optical density) ± standard deviation (SD) for triplicate samples.

Placed in inserts above A-431 cells, 10% HPMC gel (100 mg HPMC/mL) slightly decreased, by approximately 10%, the intensity of the MTS reduction in exposed samples as compared to the control (Figure 10). The precision of the results was limited by the fact that the 10% HPMC gel is difficult to pipette due to its elevated viscosity; hence, the quantity of gel put in the inserts may have varied among the samples.

When cells were suspended in 10% HPMC gel diluted in 2D culture medium for handling the gel more precisely and for suspending the cells, the intensity of the MTS reduction was decreased by approximately 40% of the untreated control (Figure 10); the HPMC polymers apparently became more toxic when the cross-linking of the polymers was altered by mechanical disruption of the gel in the culture medium.

**Figure 11:**
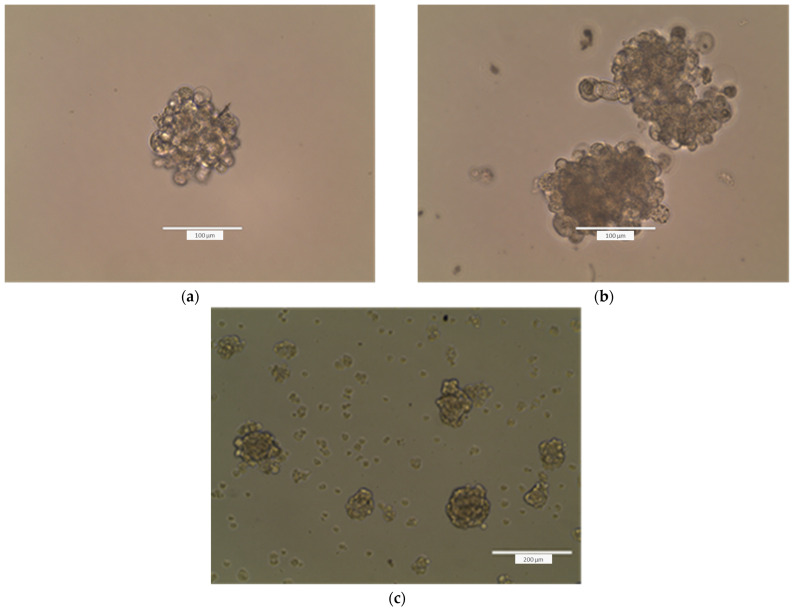
Images of A-431 epidermoid carcinoma cells seeded over a layer of 10% HPMC gel at 24 h after seeding (**a**) and further visualized at 5 days after gel removal and cultivation of cells in 3D culture medium (**b**). Images were taken with an EVOS 200 inverted microscope. (**c**) Images of HaCaT keratinocytes seeded over a layer of 10% HPMC gel, visualized at 24 h after seeding with an EVOS 200 inverted microscope.

Microscopic observation of A-431 cells layered on the top of the HPMC gel at 24 h showed that cells migrated into the gel and formed spheroids (Figure 11a). Most of the spheroids were formed at the bottom of the gel. The gel and culture medium were removed, and the 3D culture medium was placed in each well. More spheroids were formed 24 h thereafter (Figure 11b). The HPMC also favored the formation of spheroids in the case of human normal HaCaT keratinocytes (Figure 11c). The results indicate, first, that the gel is biocompatible with the investigated cell lines and that the gel sustains the formation of 3D cultures.

#### 2.3.2. PS Uptake by Cells Formulated in HPMC Gels

**Figure 12:**
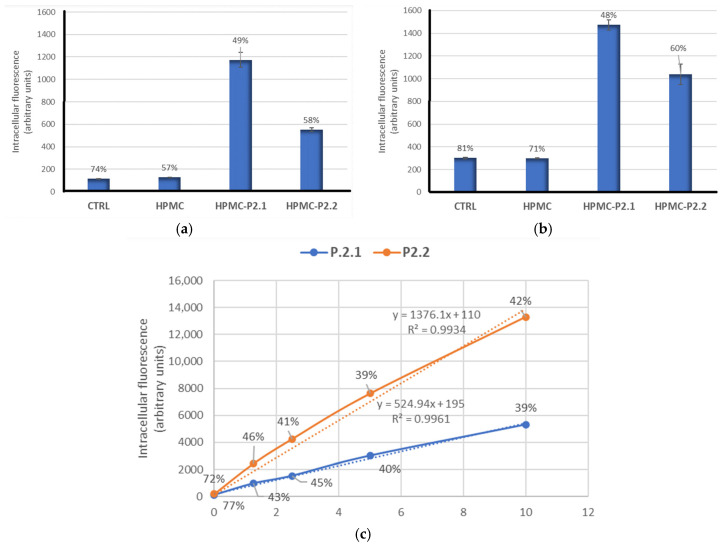
Uptake of P2.2 and P2.1 formulated in 10% HPMC gel (with 400 µM porphyrin) by A-431 human epidermoid carcinoma cells: (**a**) experiment 1; (**b**) experiment 2; (**c**) concentration–uptake curves for soluble PS. The dash line represents the linear trendline.

The uptake of the investigated porphyrinic photosensitizers (P2.1 and P2.2) formulated in HPMC gel (with 400 µM porphyrin) by human A-431 epidermoid carcinoma cells was analyzed by flow cytometry, taking advantage of the fluorescent properties of these compounds (Figure 12a,b) [10,27]. HPMC-P2.1 and HPMC-P2.2 were placed in inserts on top of adhered cells and were allowed to release PS for 24 h. PS uptake was measured by flow cytometry based on the fluorescence emission of porphyrinic PS in the FL3 channel (red). The mean fluorescence intensity (geomean) ± SD values are represented, along with the coefficients of variation (CV%) placed above the columns. Triplicate samples were analyzed. Therefore, it is expected that the accumulation of PS in normal and tumor skin cells may be significantly higher when PS-containing gels are placed directly on the skin.

A trend toward higher incorporation of P2.1 compared to P2.2 was observed, allowing variability between experiments. Based on the concentration–uptake curve presented in Figure 12c, the intracellular concentration of P2.1 in A-431 cells was 1.8 µM in experiment 1 and 2.4 µM in experiment 2. The intracellular concentration of P2.2 was 0.32 µM in experiment 1 and 0.68 µM in experiment 2. It can be noted that P2.1 and P2.2 released from HPMC gels placed in inserts were diluted in 700 µL of 2D culture medium.

Of note is that the variability between experiments is mainly due to the viscosity of the HPMC gel, which does not allow precise pipetting. Accordingly, these are only qualitative results showing that PS formulated in HPMC gel is incorporated into skin carcinoma cells and makes them sensitive to PDT. 

The porphyrin derivatives P2.1 and P2.2 formulated in HPMC gel matrices demonstrate PS uptake and subsequent internalization into human epidermoid carcinoma cells. The gel matrix is only slightly cytotoxic for tumor cells, but the disruption of polymer cross-linking may increase cytotoxicity. These findings show that the HPMC gel sustains the formation of spheroids, being suitable for in vitro studies on tumorspheres that better resemble real tumors than 2D cell cultures.

## 3. Conclusions

In conclusion, this research successfully confirms the effective incorporation of the two porphyrin derivatives into hydroxypropyl methylcellulose (HPMC) hydrogels, highlighting their potential as advanced delivery systems for topical photodynamic therapy (PDT). HPMC not only demonstrated a stable and biocompatible matrix but also contributed to improving key formulation characteristics. Its intrinsic film-forming ability, bioadhesiveness, and controlled swelling behavior enhanced the mechanical stability, spreadability, and moisture retention of the hydrogel formulations. The inclusion of porphyrins influenced the rheological profile of the HPMC gels, inducing changes in viscosity and shear stress—indicative of specific interactions within the polymer network. The pH remained within the optimal range for dermal use, minimizing irritation risks. Overall, the use of HPMC not only preserved but also improved the functional and physicochemical properties of the porphyrin-loaded hydrogels, supporting their potential for effective use in topical PDT. Further research will explore the optimization of HPMC-based porphyrin gels, their long-term stability, and their potential combination with other therapies to enhance topical photodynamic treatment of skin cancer.

## 4. Materials and Methods

### 4.1. Materials

The studied porphyrins were obtained following the methods previously outlined by our team [10]. Polyethylene glycol 200 (PEG 200) and hydroxypropyl methylcellulose (HPMC) were acquired from Sigma-Aldrich Chemie GmbH, Taufkirchen, Germany. All substances were measured using a Mettler Toledo AT261 balance, which has a sensitivity of 0.01 mg.

### 4.2. Methods

#### 4.2.1. Hydrogel Formulation and Manufacturing

HPMC is a non-ionic polymer with a linear glucose molecular structure, and hydrogen bonds stabilize its matrix. Considering its high biocompatibility and its ability to form stable and bioadhesive hydrogels [69,70,71] it was chosen as a gel-forming polymer. Using a Heidolph MR 3001K magnetic stirrer (Schwabach, Germany), HPMC was dissolved in water and mixed at 800 rpm at room temperature to obtain a 10% HPMC hydrogel. The hydrogel was cooled at 5 °C throughout the night to allow deaeration. The produced gel functioned as a foundation for the porphyrin gels under study as well as a point of reference in the ensuing investigations.

A quantity of 50 g of the base gel (10% HPMC) was mixed at room temperature and at 750 rpm, in the dark, with a 10 mM porphyrin solution in PEG 200 to obtain a hydrogel formula with a content of 0.3 mg porphyrin/g gel. The formed gel’s characteristics were assessed in comparison to the base 10% HPMC hydrogel.

#### 4.2.2. Physicochemical Characterization of the Gels

Fourier-transform infrared (FTIR) spectra were obtained using a JASCO FTIR 4100 spectrophotometer (Tokyo, Japan) in the range of 4000–400 cm^−1^, recorded as transmittance vs. wavenumbers.

X-ray diffraction (XRD) analysis was carried out on a Rigaku Ultima IV diffractometer (Rigaku Co., Tokyo, Japan) equipped with CuKα radiation (λ = 1.5406 Å) in parallel beam geometry. Diffractograms were collected over a 2θ range of 5°–80°, with a scanning speed of 2°/min and a step size of 0.02°.

Thermogravimetric analysis (TGA) was performed on a Mettler Toledo TGA/SDTA851^e^ instrument (Mettler-Toledo, Greifensee, Switzerland) under synthetic air flow (80 mL/min) from 30 °C to 600 °C at a heating rate of 10 °C/min to evaluate thermal decomposition behavior.

Atomic force microscopy (AFM) analyses were conducted using the XE-100 system (Park Systems, Suwon, Republic of Korea) operating in non-contact mode. All measurements were carried out with NSC36B tips (MicroMasch, Neuchatel, Switzerland), which have a curvature radius under 8 nm, an approximate cone angle of 40°, a height of around 15 μm, a thickness of roughly 1 μm, a length of about 90 μm, and a width near 32 μm. These probes also have a force constant of approximately 2 N/m and a resonance frequency close to 130 kHz. The obtained AFM images were displayed using an “enhanced contrast” setting, where pixel coloration is influenced by the surrounding pixel values to improve visual differentiation. For surface analysis, a small droplet from the obtained hydrogel-based porphyrins was placed onto a clean microscope glass slide (Heinz Herrenz Medizinalbedarf GmbH, Hamburg, Germany) and allowed to dry at room temperature. Image processing was performed using the XEI software (v1.8.0, Park Systems), primarily for tilt correction and presentation. Surface roughness was evaluated using several parameters: (i) from the amplitude parameters, the root mean square roughness (Rq), defined as the standard deviation of the surface height value, and the peak-to-valley parameter (Rpv), representing the height difference between the lowest and the highest surface points, were determined; (ii) the functional parameters included reduced summit height (Spk—the height of the material in the peak zone), core roughness depth (Sk—the height difference between the intersection points of the found least mean square line), and the reduced valley depth (Svk—the height of the valley zone).

#### 4.2.3. UV-Vis and Fluorescence Studies

UV-Vis spectral measurements were performed using a LAMBDA 35 UV/Vis spectrophotometer (Perkin Elmer Life & Analytical Science^®^, Waltham, MA, USA) equipped with a deuterium and tungsten light source with automatic switching to ensure high precision and reproducibility. Absorption spectra were recorded using the single-cell holder containing hermetically sealed quartz-coated optical mirrors and a lens-free system to minimize measurement error. Both absorption and fluorescence spectra of gel samples containing porphyrins were obtained using 1 cm quartz cuvettes. Fluorescence measurements were recorded using the JASCO FP 6500 spectrofluorometer (JASCO Co., Ltd., Kyoto, Japan) with an excitation wavelength of 426 nm, in accordance with the UV-Vis spectra of the porphyrin-containing gels.

#### 4.2.4. Pharmacotechnical Characterization of the Porphyrin Gels

Wet gel evaluation

##### pH Determination

Hydrogel samples (0.2 g) were diluted in 1 mL of distilled water (pH 6.5 ± 0.5), and the pH values of each solution were measured using the electrode of a CONSORT P601 pH meter (manufactured by CONSORTnv, Turnhout, Belgium).

##### Spreadability

Spreadability was evaluated using a glass plate method. A quantity of 1 g of each hydrogel was placed at the center of a 2 cm diameter circle on a glass plate. A second glass plate with a 150 g weight was then applied to the surface, and the diameter of the spread gel was measured. After a 2 min equilibration, additional weights (50 g, 100 g, 150 g, 200 g, 250 g, 300 g, and 500 g) were successively placed on the upper glass plate, and the corresponding spread diameters were measured. The experiments were performed in triplicate. The following formula was used to determine the spreading area occupied by the hydrogel (Equation (1)):

S = πr^2^ (1)

#### 4.2.5. In Vitro Adhesion Ability

A 1 cm^3^ hydrogel layer was applied thinly on a digital tensilemeter clamp (LR 10K Plus, West Sussex, UK). The hydrogel was subsequently compressed using the second clamp. The test was performed at a speed of 100 mm/min, and the mass required to remove the second clamp from the gel surface was recorded. The tensile force necessary to remove the second clamp from the entire hydrogel-covered area was used to calculate the adhesion capacity (Equation (2)).

(2) $$Tensile\;force\;\left(\frac{g}{{mm}^{2}}\right)=\frac{Detaching\;mass\;(g)}{Surface\;({mm}^{2})}$$

#### 4.2.6. Rheology Measurements

Rheological measurements were carried out on 50 g of each hydrogel using a B-one Plus rotational viscometer (Lamy Rheology Instruments, Champagne du Mont d’Or, France) equipped with an RV7 spindle. The rotation speed was increased from 50 to 250 rpm at 22 °C and then repeated in reverse order. Each measurement was conducted for 150 s, without a pause between steps. The hysteresis curves of shear stress and dynamic viscosity were recorded.

#### 4.2.7. Dry Gel Evaluation

To determine the behavior of the hydrogels after their application to the skin, they were poured thinly into Petri dishes and dried for 24 h at room temperature (22 °C). The dried films were carefully peeled from the substrate and subsequently evaluated for their mechanical and swelling properties.

#### 4.2.8. Mechanical Properties

##### Thickness

Film thickness was measured using a digital micrometer (0–25 mm range and 0.001 mm resolution; Yato Trading Co., Ltd., Shanghai, China).

##### Tensile Strength and Elongation

The tensile strength and elongation were evaluated using a digital tensile strength tester for universal materials (Lloyd Instruments Ltd., LR 10K Plus, West Sussex, UK). The test was made at a distance of 20 mm and at a speed of 3 mm/s by aligning the films vertically between the two braces. The breaking force could be calculated [72] using the following formula:

(3) $$Tensile\;strength\left(\frac{kg}{{mm}^{2}}\right)=\frac{Force\;at\;breakage\;(kg)}{Film\;thickness\;\left(mm\right)\times Film\;width\;(mm)}$$

The following formula was used to determine the elongation at break:

(4) $$Elongation\left(\%\right)=\frac{Increase\;in\;film\;length}{Initial\;film\;length}\times 100$$

##### Moisture Content

The thermogravimetric method was utilized to evaluate the loss on drying using a HR 73 Mettler Toledo halogen humidity analyzer produced by Mettler-Toledo GmbH, Greifensee, Switzerland [73].

##### Swelling Ratio

The method used to calculate the swelling ratio involved weighing 0.2 g of the dried hydrogel-formed film incubated at 37 ± 1 °C, in Petri plates with 1.5% agar gel, every 30 min for six hours. The following formula was used to calculate the swelling ratio:

(5) $$Swelling\;ratio=\frac{Wt-Wi}{Wi}\times 100$$

where Wt is the patch mass at time t after the incubation and Wi is the initial mass [74,75,76].

#### 4.2.9. In Vitro Study

Considering that HPMC gels containing porphyrin photosensitizers (P2.1 and P2.2) were designed for local PDT in skin carcinoma, a preliminary in vitro study was performed on the A-431 cell line (human epidermoid carcinoma) to evaluate the biocompatibility of the HPMC gel and the uptake of PSs released from the gel.

The main issue was to handle the viscous HPMC gel and the recovery of cells treated with the gel. The experimental methods used in the present in vitro study were described in [27].

#### 4.2.10. Gels and Porphyrin Compounds

The porphyrin compounds P2.1 and P2.2, formulated in HPMC gel or in PEG 200, were analyzed. P2.1 and P2.2 were dissolved in PEG 200 at a concentration of 10 mM. Then, 10% HPMC base gel and gels containing PSs, either P2.1 or P2.2, at a concentration of 400 µM (HPMC-P2.1 and HPMC-P2.2) were analyzed. PS solutions and gels were stored at room temperature, in the dark, until use. For specific biological tests, the gel was weighed, and culture medium was added to dilute the gel mechanically by vigorous pipetting.

#### 4.2.11. Cells

Human skin cell lines, either tumoral (A-431 epidermoid carcinoma cell line; ATCC, Manassas, VA, USA) or normal cells (HaCaT keratinocytes; CLS Cell Lines Service GmbH, Eppelheim, Germany) were investigated. Cells were maintained in 2D cultures in DMEM-F12 medium (Gibco, Thermo Fisher Scientific, Waltham, MA, USA) supplemented with 10% fetal bovine serum (FBS, Sigma, Saint Louis, MO, USA) and antibiotic–antimycotic solution (Sigma, Burlington, MA, USA), further nominated as complete 2D culture medium. Cell passage was performed 2–3 times per week using Trypsin/EDTA 0.05%/0.02% (PAN Biotech GmbH, Aidenbach, Germany). DMEM-F12 medium (Gibco, Thermo Fisher Scientific, Waltham, MA, USA) supplemented with 2% FBS (Sigma, Saint Louis, MO, USA) and antibiotic–antimycotic solution (Sigma, Burlington, MA, USA), further nominated as loading culture medium, was used for loading cells with P2.1 or P2.2 dissolved solely in PEG 200. For obtaining tumorspheres, 3D culture medium (3D Tumosphere Medium XF medium, PromoCell, Heidelberg, Germany) supplemented with 1% antibiotic–antimycotic solution (Sigma, Burlington, MA, USA) was used alone or in combination with HPMC.

#### 4.2.12. Biocompatibility of HPMC Gel

We used two settings for exposing cells to HPMC gel: (i) A total of 7 × 10^4^ A-431 cells were cultivated in 700 µL complete 2D culture medium in 24-well plates for 18 h to allow cells to adhere to the culture plates. Thereafter, the HPMC gel was added in Falcon inserts with 0.4 µm Transparent PET Membrane (Greiner, Kremsmünster, Austria) to obtain an approx. 2 mm thick layer of gel at the bottom of the insert, and cells were further cultivated for 24 h. A blank insert was used in the case of the control sample. This experimental setting provides information on the indirect interaction of cells with the gel. (ii) The 10% HPMC gel, put in 2D culture medium (0.325 g HPMC gel and 0.65 mL 2D culture medium), was vigorously homogenized and was diluted in 2D culture medium (1/8–1/4). Cells were suspended in this easy-to-handle HPMC form. Of note is that gas bubbles were formed during the homogenization process. This experimental setting provides information on the direct interaction of cells with HPMC polymers with disturbed cross-linking.

#### 4.2.13. The MTS Reduction Test

In the experimental settings (i) and (ii), described above, the biocompatibility of HPMC was evaluated by the tetrazolium salt reduction test (MTS, 3-(4,5-dimethylthiazol-2-yl)-5-(3-carboxymethoxyphenyl)-2-(4-sulfophenyl)-2H-tetrazolium) using the CellTiter 96^®^ AQueous One Solution Cell Proliferation Assay kit (Promega Corporation, Madison, WI, USA). This method provides information on the number of metabolically active cells in culture. Briefly, A-431 cells exposed to HPMC or only to 2D culture medium (control) were detached from the bottom of the cell culture plates with Trypsin/EDTA 0.05%/0.02% (PAN Biotech GmbH, Aidenbach, Germany), washed with 2D culture medium by centrifugation, and counted, and cellular viability was measured using the Trypan blue exclusion test. Cells were suspended in a total volume of 100 µL of complete culture medium in 96-well plates (10,000 cells/100 µL/sample), 20 µL of the MTS kit reagent was added, and samples were incubated at 37 °C in a 5% CO_2_ atmosphere for 90 min. Optical densities (ODs) were measured using an ELISA reader (Tecan, Männedorf, Switzerland) at 490 nm, compared to a reference wavelength of 620 nm. ODs were obtained with the Magellan standard software (Tecan Life Sciences, Männedorf, Switzerland). The ODs of the test samples were corrected by subtracting the mean OD for the background sample (samples that contained only culture medium with or without HPMC and did not contain cells). The results were expressed as the mean corrected OD ± standard error of the mean (SEM) for triplicate samples. Of note is that dilutions of HPMC (1/24 and 1/12) used in some experiments did not affect the background readings.

#### 4.2.14. Formation of Tumorspheres

An A-431 carcinoma cell suspension (0.5 × 10^6^ cells/500 µL 3D culture medium) was layered over a bed of HPMC gel in 12-well plates, and cells were cultivated for 24 h. The gel and culture medium were then removed, and 0.5 mL 3D culture medium was added in each well. Cells were visualized by optical microscopy using an EVOS 200 inverted optical microscope (Thermo Fischer Scientific, Hillsboro, OR, USA) at 24 h after seeding and at 5 days after gel removal and cultivation of cells in 3D culture medium.

#### 4.2.15. P2.1 and P2.2 Uptakes

For investigating the uptake of P2.1 and P2.2 formulated in HPMC gels, 7 × 10^4^ cells were first cultivated for 18 h in 700 µL loading culture medium. Thereafter, the P2.1- or P2.2-containing HPMC gels were added in Falcon inserts with a 0.4 µm Transparent PET Membrane (Greiner, Kremsmünster, Austria) to obtain a 1–2 mm thick layer of gel. Cells were further cultivated for 24 h to allow PS release from gels and their incorporation into cells. Finally, adherent cells were detached from the bottom of the plate with Trypsin/EDTA 0.05%/0.02%. Detached cells were washed by centrifugation with phosphate-buffered saline (PBS) and were finally fixed with 4% paraformaldehyde (Fixation buffer, BioLegend, San Diego, CA, USA). PS uptake was measured by flow cytometry, taking advantage of the fluorescent properties of the investigated PS [77].

The fluorescence emission of intracellular PSs was measured in the FL3 fluorescence channel (red) for at least 5000 cells/sample using a BD FACS Canto cytometer (Becton Dickinson, Franklin Lakes, NJ, USA) equipped with a 488 nm laser. Data were processed with the BD FACSDiva software (Becton Dickinson, Waltham, MA, USA), and results were expressed as the geometric mean of the fluorescence intensity expressed in arbitrary units. The coefficient of variation (CV%), characterizing the dispersion of the fluorescence intensity values around the mean value, was also represented on graphs.

The uptake of soluble P2.1 and P2.2 dissolved in PEG 200 by A-431 tumor cells was used as a reference for approximating the release of PS from HPMC gels. Briefly, 7 × 10^4^ cells were cultivated in 500 µL complete 2D culture medium in 24-well plates for 18 h to allow cells to adhere to the culture plates. The culture medium was replaced with 700 µL of loading culture medium containing P2.1 or P2.2, and cells were cultivated for another 24 h to allow PS incorporation into cells. Various concentrations of PSs were investigated to obtain a dose–uptake curve.

## 5. Patents

a. Patent application a 2024 00567: E. A. Ozon, A. M. Burloiu, R. Boscencu, G. Manda, V. Anuta, C. E. Dinu Pirvu, D. Lupuliasa, I. V. Neagoe, A. M. Musuc, M. Anastasescu, R. P. Socoteanu, Asymmetric porphyrin in hydroxypropylmethylcellulose matrix for the treatment of premalignant skin disorders, published in RO-BOPI, 2 from 28 February 2025.

## Figures and Tables

**Table 1:** The thermal data obtained from TGA curves.

Gels	1st Step (Temperature and Mass Loss)	2nd Step (Maximum Peak Temperatures and Mass Loss)	Remaining Mass at 600 °C
10% HPMC	Below 100 °C/2.7%	*T*_DTA_ = 335 °C and *T*_DTG_ = 330 °C *T*_DTA_ = 461 °C and *T*_DTG_ = 458 °C	0.38%
HPMC-P2.1	Below 100 °C/2.2%	*T*_DTA_ = 342.16 °C and *T*_DTG_ = 345 °C *T*_DTA_ = 453.8 °C and *T*_DTG_ = 453.5 °C	No residue
HPMC-P2.2	Below 100 °C/2%	*T*_DTA_ = 342.6 °C and *T*_DTG_ = 346.5 °C *T*_DTA_ = 447 °C and *T*_DTG_ = 443 °C	No residue

**Table 2:** Spectral characteristics of porphyrins P2.1 and P2.2 in HPMC gels.

	Absorption λ*_max_* (nm)					Emission λ_max_ (nm)
	Soret Band	Qy(1,0)	Qy(0,0)	Qx(1,0)	Qx(0,0)	
HPMC-P2.1	426	518	552	591	658	654
HPMC-P2.2	428	521	556	594	652	656

## Data Availability

Data are contained within the article.
